# Fabrication and Performance Evolution of AgNP Interdigitated Electrode Touch Sensor for Automotive Infotainment

**DOI:** 10.3390/s21237961

**Published:** 2021-11-29

**Authors:** K. P. Srinivasan, T. Muthuramalingam

**Affiliations:** Department of Mechatronics Engineering, SRM Institute of Science and Technology, SRM Nagar, Kattankulathur, Chennai 603203, India; sp3048@srmist.edu.in

**Keywords:** integrated electrode, printed capacitive sensor, infotainment bezel, seamless instrument panel, screen printing

## Abstract

In the present scenario, a considerable assiduity is provided to develop novel human-machine interface technologies that rapidly outpace the capabilities of display technology in automotive industries. It is necessary to use a new cockpit design in conjunction with a fully automated driving environment in order to enhance the driving experience. It can create a seamless and futuristic dashboard for automotive infotainment application. In the present study, an endeavor was made to equip the In-vehicle bezels with printed capacitive sensors for providing superior sensing capabilities. Silver Nanoparticles based interdigitated pattern electrodes were formed over polycarbonate substrates to make printed capacitive sensors using screen printing process. The developed sensor was investigated to evaluate the qualitative and quantitative measures using direct and in-direct contact of touch. The proposed approach for sensors pattern and fabrication can highly impact on sensor performance in automotive infotainment application due to the excellent spatial interpolation with lower cost, light weight, and mechanical flexibility.

## 1. Introduction

**Figure 1:**
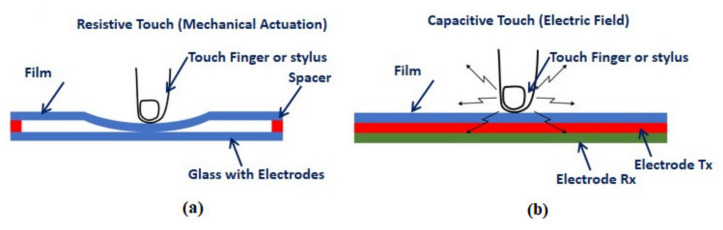
Working mechanism of touch panel sensors (**a**) Resistive (**b**) Capacitive.

Integration of automotive electronics into a vehicle plays a critical role in the advancement of vehicle performance, safety, and ergonomics in order to meet and exceed the expectations of the customer [1]. The replacement of traditional mechanical buttons and knops with flexible touch panels should receive significant attention in cockpit electronics in order to attain a futuristic look and feel while also improving passenger convenience [2]. When used in conjunction with a lightweight flexible electronics system, the human machine interface (HMI) can be reduced in weight, which is beneficial in automobile vehicles. Printed interdigitated electrode (IDE) based Capacitive Sensor (PICS) are becoming increasingly popular within the automobile industry [3], owing to their higher production rates, flexibility, and ability to accommodate complex geometries. In order to achieve seamless cockpit integration [4], such flexible sensors may be easily created utilizing screen printing technology, which has lower production costs while also providing good formability to create three-dimensional (3D) forms. As a result, flexible printed sensors are particularly well suited for use in In-Mold Electronics (IME) based infotainment bezel applications [5,6]. Through the IME method, the In-Vehicle Infotainment System (IVI) bezel may be simply produced into 2D and 3D forms in accordance with the ECE21automotive standard [7]. This type of sensor can be divided into two categories: resistive touch principles and capacitive touch principles [8]. Due to the fact that the resistive touch panel does not enable multi touch gestures and has no IME compatibility, it is recommended that the capacitive based flexible touch sensor be used for the infotainment bezel application rather than the resistive touch panel. The resistive touch panel works under the principle of mechanical actuation, however the capacitive touch works under the mechanism of electric field change as shown in Figure 1. Hence the capacitive touch panel has been proposed for the mentioned application.

Insulated plastic sheet with conductive ink materials can be used to construct capacitive-based touch screen sensors for use in mobile devices. The construction of such sensor is possible with Inkjet printing [9,10], however it is limited due to its time and ink material and also it may be used to check for proto typing and low volume production of sensor. For Automotive screen-printing process is widely used and it is possible to create a conductive ink pattern either by using a continuous conductive line path pattern (or) by using two isolated conductive electrodes based interdigitated patterns to create conductive ink. Two conductive ink pattern electrodes are used to construct these Printed Integrated electrodes Capacitive Sensor, which are separated by a dielectric medium. Because of their superior physical and electrical qualities, silver nano particles (AgNP) conductive materials can give better sensitivity with lower wear rate among all conductive materials for creating Printed Integrated electrode Capacitive Sensor (PICS) in IVI bezel application compared to other conductive materials [11,12]. 

In order to create the final infotainment product, the flexible printed conductive layers are developed over the hard plastic layer. Considering that capacitance is directly proportional to electrode overlap area, spatial interpolation of electrodes structure in capacitive touch panels has a substantial impact on sensor performance [13,14], it is important to consider how the electrodes are organized. Due to its ability to change the capacitance, the distance between electrode structures can also have an impact on the sensitivity of an electrode structure. It is critical to pick the ideal overlap area and distance between electrodes in order to achieve improved sensitivity while also lowering production costs [15,16]. Digital mobility, increasing volumes of information [17,18], and drivers’ lives that are becoming increasingly linked and mobile, call for new methods of transmitting information, which are quickly reaching the limitations of display technology and operating system capabilities. A completely automated driving environment must also be used in conjunction with the new cockpit design in order for it to be successful. It is vital to have an understanding of future automotive advances.

## 2. Proposed Methodology

**Figure 2:**
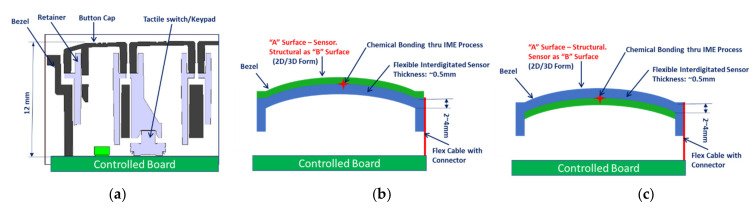
Configuration of IVI Bezel (**a**) Current Design (**b**) Proposed—Direct Touch (**c**) In-Direct Touch.

**Figure 3:**
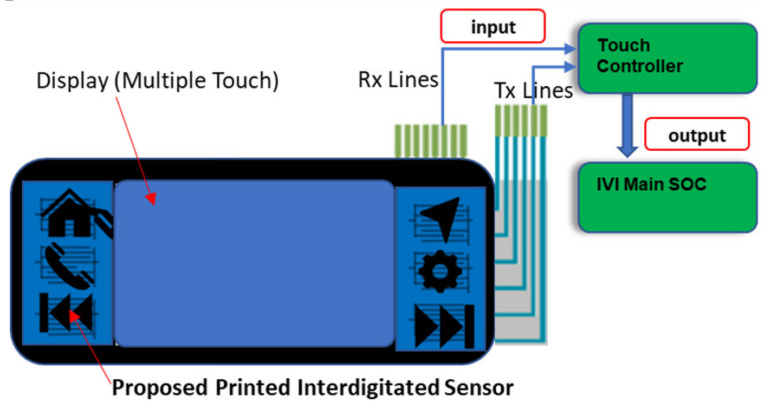
IME bezel with flexible capacitive sensor Block Diagram.

The Conventional Buttons, which are comprised of a button cap, a retainer, a tactile switch, a hard printed circuit board (PCB), and a display arrangement, make the device larger and heavier in comparison to other options and sensor can be thermo-formed into 3D surface [19,20]. Using an IDE Printed sensor in conjunction with an OGS Display, it may be able to create a seamless and lightweight infotainment system for the automotive cockpit. In the context of conventional mechanical tactile buttons for infotainment display panel in automotive design, the printed capacitive touch sensors were proposed as shown in Figure 2. Thestyling surfaces are classified as Class “A” and “B” surfaces. All visible outside surfaces and all visible surfaces of see-touch-feel surfaces in the interior are classed as Class A. This classification is derived from industrial design using scanned data. All other surfaces are classified as Class B. The Class A consists of the Aesthetic appearance, textured surface. However, it does not have any characteristics such as ribs, snaps, or bosses with continuous curvature surfaces. The Class B surfaces include Invisible surfaces with devoid of textures. It includes all mounting and stiffening elements such as Rib, Boss, and Snap over the B-side surface. The sensors were created by the use of a screen-printing procedure. Figure 2 depicts a comparison of the schematic arrangement of screen printing for printed flexible electronics and mechanical tactile buttons that are used in traditional mechanical devices. It is possible to attach the printed flexible sensor in one of the following ways: Option1: Sensor is on TOP for IVI Bezel as a Class “A” Surface Option2: The sensor is located at the bottom of the IVI bezel as “B” Surface. Option 2 is better because it prevents wear and tear of the sensor pattern caused by direct contact with the driver. Due to the suggested printed sensor technique, the weight of the infotainment system can be decreased significantly. The replacement of keys in the IVI bezel for infotainment applications design and fabrication of a PICS based interdigitated pattern-based key were carried out in the current work. Due to spatial interpolation, the electric field changes when the passenger or driver presses the capacitive key, as illustrated in Figure 3.

## 3. Design and Simulation of Printed IDE Capacitive Sensor

**Figure 4:**
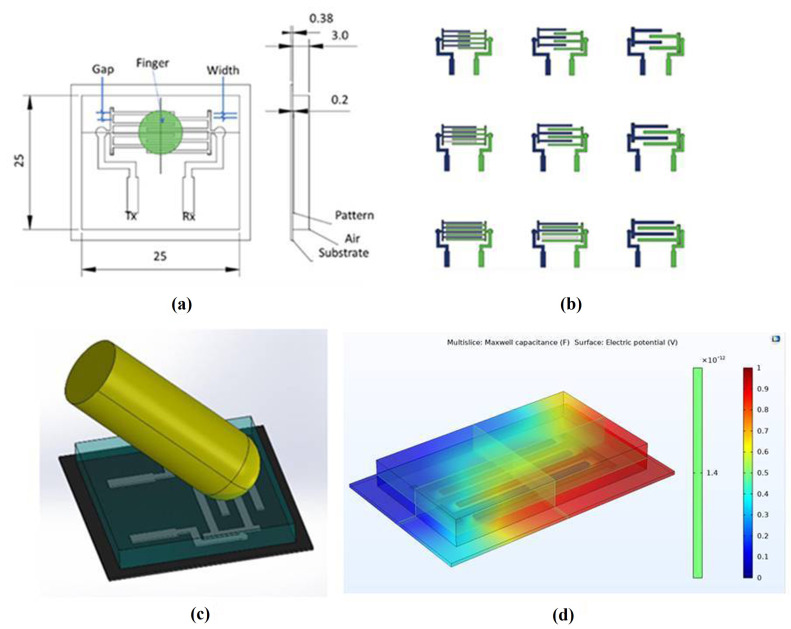
Design and simulation (**a**) Electrode Design (**b**) Sensor Pattern design (**c**) Parametric CAD Model (**d**) Capacitance/Electric Potential.

The design of an interdigitated pattern-based Printed capacitive touch panel with a 25 mm × 15 mm surface area [17] have been designed as illustrated in Figure 4 for button function. The function can be mapped electronically to mother controller with any infotainment feature such as Home, Forward, Setting, Phone, or Navigation, etc. The button sensor design was done using parametric Modeling and used CATIA CAD Modeling software. Nine trial specimens were chosen because the experimental included two input factors, overlap distance (5 mm, 10 mm, 15 mm) and electrode line width (0.5 mm, 0.8 mm, 1.2 mm), as well as three different levels. All specimens had the same line gap and coating thickness.

The simulations were performed using COMSOL Multiphysics version 5.5 to determine how the geometry (Overlap and Line width) of interdigitated electrodes affects touch sensitivity. The CATIA parametric model was converted into STEP file. The STEP format (Standard for the Exchange of Product Data) was intended to improve file compatibility between software packages so designs could be exchanged and modified more readily. The results of these simulations were then compared to the results of the experiments. The modeling of pattern with Fingers is shown in Figure 4c. The capacitance between the transmitter (T_x_) and receiver (R_x_) electrodes, which changed as a result of the introduction of a finger between the two electrodes, was measured. The simulation was run again with all nine sensors positioned in the center of the button as shown in the illustration. It displays the data that was simulated. The change in capacitance was observed both with and without the finger being used. In terms of parasitic capacitance (measured without touch), there is a strong correlation with the actual sensor measurement. Figure 4d shows the parasitic capacitance and voltage potential field.

## 4. Fabrication of Printed IDE Capacitive Sensor

**Figure 5:**
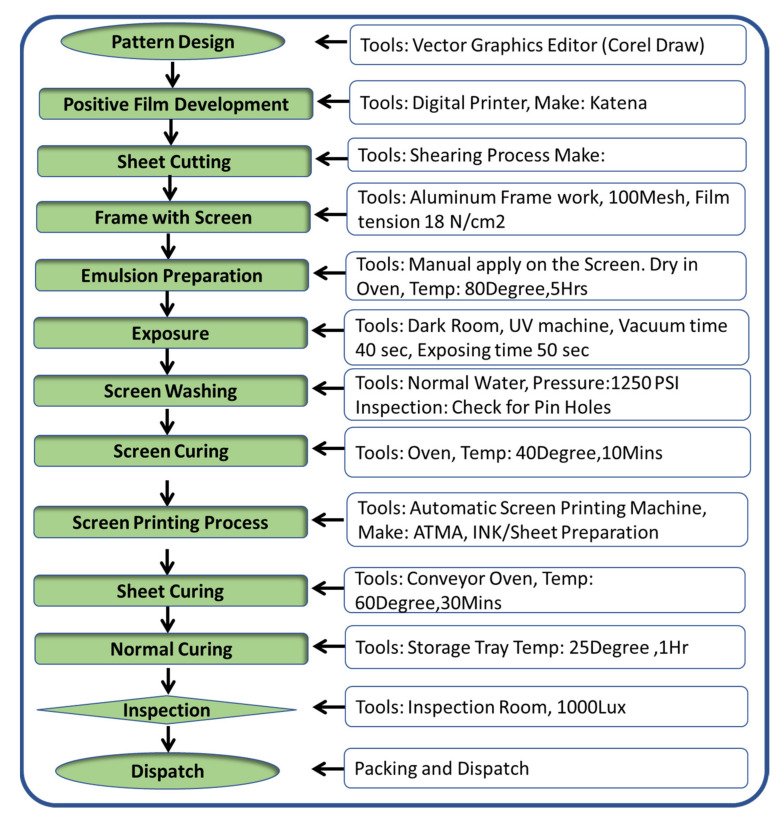
Steps involved in sensors fabrication process flow.

As part of this project, Ag nano particles conductive ink that consists of 70% Solids and 30% thermoplastics/additives (purchased from Siltech Corporation Inc, Bengaluru, India) was employed to create sensors electrodes for use in the fabrication of PICS [21,22,23]. A Polycarbonate sheets with a thickness of 375 microns was coated with a 15micronmeter layer of thickness over two separated conducting electrodes (X and Y). The sheet resistance was computed using 4 probes Hall Effect measuring system and it was found as 0.14 ohms. The fabrication process is explained in the process flow diagram as shown in Figure 5.

The electrical conductivity and opaque properties of the Ag ink particles were important considerations in their selection. The Lexan 8010 Polycarbonate sheet (produced by Sabic Innovative Plastics in the United States) was selected as the plastic substrate for IME because of its superior thermal resistance and thermoform ability, as well as its higher transparency. The Ag paste was put on top of a mesh screen frame that was coated with a photo-emulsion coating (Saio Screen frame from India). According to Equation (1), the overlapping area of the electrode (A) and the line width (B) of the electrodes are important factors for the printed capacitive sensors to be successful as per Equation (1).

(1) $$C=\;\frac{\varepsilon A}{D}$$

where c denotes capacitance in Farads; denotes electrical permittivity in Farads per meter; A denotes capacitance area in square meters; and D is the distance between electrodes in meters. Figure 5 illustrates the various stages of the design and fabrication of a Printed IDE Capacitive Sensor, including the design phase, the fabrication phase, and the testing phase. 

**Figure 6:**
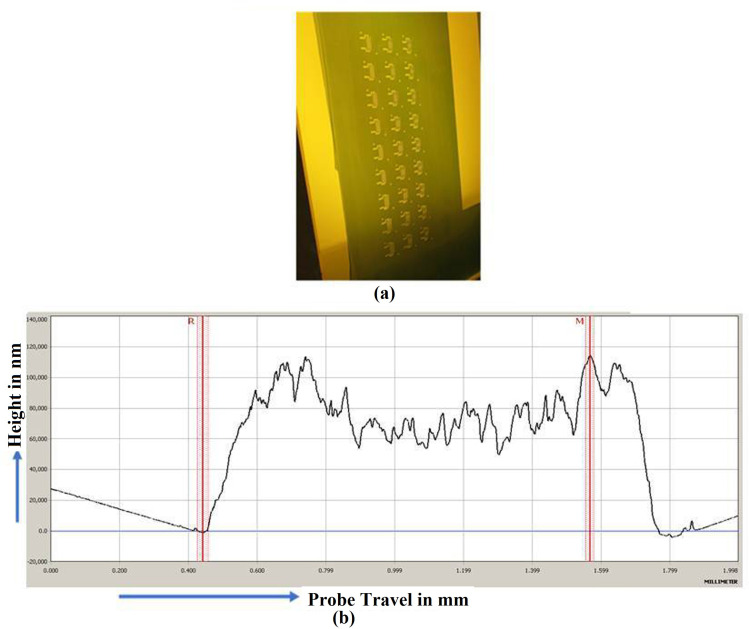
Fabrication and Coating measurement (**a**) Exposing in Dark room (**b**) Coating thickness chart acquired using stylus profiler.

During the fabrication process, the screen was used in the exposing phase that took place in a dark room. Following that, the printing procedure was carried out using an ATMA screen printer machine. The sheets were then exposed to the curing process for approximately 30 min in both the oven and at room temperature. The Coating thickness is measured precisely using KLA Tencor Alpha-Step D500 Stylus Profiler coating thickness as shown in Figure 6. After that, a quality check was carried out using a light inspection meter with a reading of less than 1000 Lux.

## 5. Performance Evaluation of Sensor

The TTP223 is an integrated circuit that detects the presence of a touch pad and provides one touch key. To be used in place of the standard direct button key, the touching detection IC is intended to work with a variety of different pad sizes. 

### 5.1. Verification of Printed Sensors 

**Figure 7:**
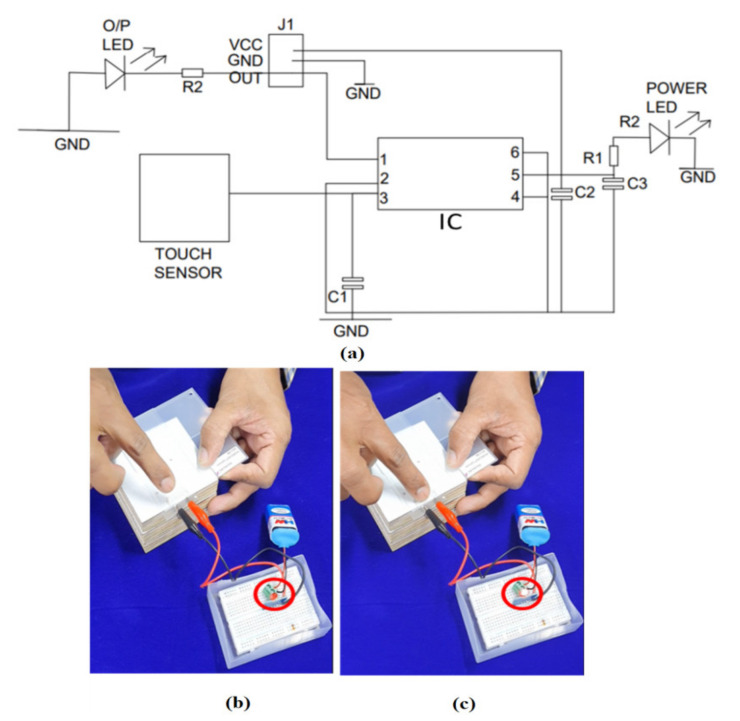
Demonstration of PICS with IVI Bezel (**a**) Circuit Diagram (**b**) Sensor with LED “OFF” (**c**) Sensor with LED “ON”.

The electronic components, such as the Touch IC, jumpers, wires, battery, and breadboard, are purchased from the open market and connected in the manner depicted in the circuit diagram as shown in the Figure 7. Two clips are used to connect the touch sensor to the breadboard assembly (T_x_ as Red and R_x_ as Black). Both C1 and the sensor are connected in parallel and calibrated to have equal capacitance values. When a touch is detected, the capacitance is reduced in accordance with the CMOS output. The output of which is converted into the state of an LED on or off. The sensor’s functionality was evaluated qualitatively, and it was discovered that the LED turned on when a finger was placed on the sensor.

### 5.2. Pattern Accuracy Test

**Figure 8:**
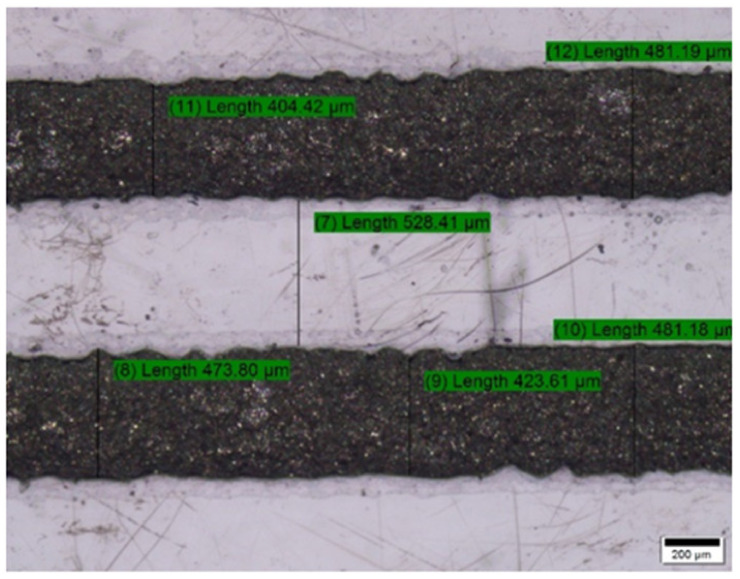
0.5 mm Electrode profile of Printed sensors characterization using optical microscope.

The fabricated electrode has been characterized for checking pattern accuracy of Electrode profile using Olympus BX51 optical microscope (OPM) as shown in Figure 8. The smooth edges with 0.5 mm of thickness have been observed in the pattern. The surface morphology and accuracy can alter the performance measures of any system and product. It can be analyzed using OPM [24,25]. However, many scratch marks on substrate could have been observed owing to the handing issues from different stage of development. These marks can be eliminated with careful handling. 

### 5.3. Adhesive Test on Sensors 

**Figure 9:**
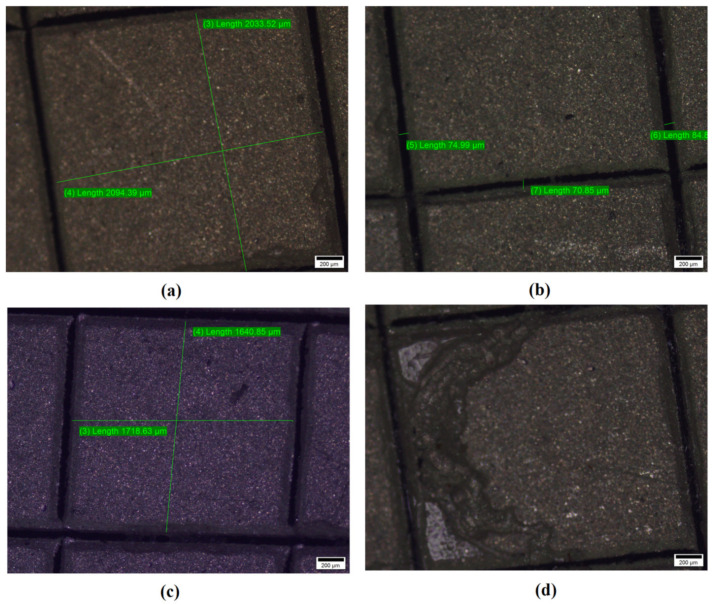
Specimen and tools Simulation (**a**) Grid (2 × 2 mm) (**b**) Grid verification (**c**) Depth of cut Verification (**d**) Adhesive on Coating.

The adhesion tests have been carried out at room temperature in order to determine the levels of adhesion between the silver Electrodes of the sensor and the substrate (Polycarbonate). The ISO 2409 standard, as well as comparisons between several Automotive OEM (Original Equipment Manufacturer) standards, where a grid of ten square cuts, spaced 2 mm apart, is used to pull out the tape at a 90-degree angle. The grid cut was produced with a knife on a silver layer that was approximately 15 micrometers thick as shown in Figure 9 and had previously been screen printed on the substrate and fabricated along with the other sensor designs on the same substrate. In order to eliminate variations due to multiple set-ups, the sensor design and Adhesion test specimen were fabricated in a single set-up. The adhesive tape 555, 25 mm wide from wonder (transparent) was applied to the cuts with uniform pressure applied with the fingers, and then it was carefully removed without jerking. The results might be classified according to the scale of the standard based on a simple visual inspection. Example of a score of 0: There is no separation or cracking of the square. As a result, adhesion conforms to the Score 0 of ISO standard. Figure 9 depicts optical image samples, where the cut depth is ensured to be sufficient to ensure that the coatings are entirely curled. It has been observed that the adhesive from the tap is transmitted to the coating, indicating that the coating is far stronger than the bond between the tape and the adhesives.

## 6. Sensitivity Measurement of Fabricated Sensor

**Figure 10:**
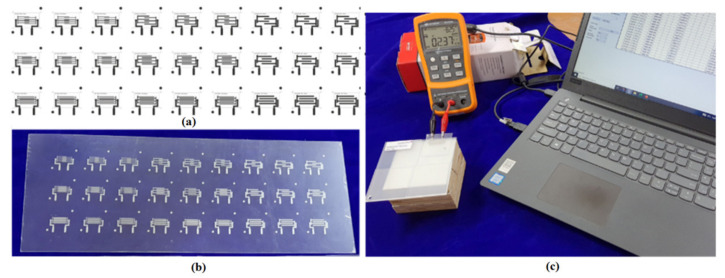
Design to Performance stage (**a**) Design of pattern (**b**) Fabricated sensors pattern (**c**) Change in capacitance measurement while touched.

The TTP223 is an integrated circuit that detects the presence of a touch pad and provides one touch key. To be used in place of the standard direct button key, the touching detection IC is intended to work with a variety of different pad sizes. The electronic components, such as the Touch IC, jumpers, wires, battery, and breadboard, are purchased from the open market and connected in the manner depicted in the circuit diagram as shown in the Figure 10 [26]. The various design patterns in different changes in the two parameters with three different variables as shown in Table 1.

**Figure 11:**
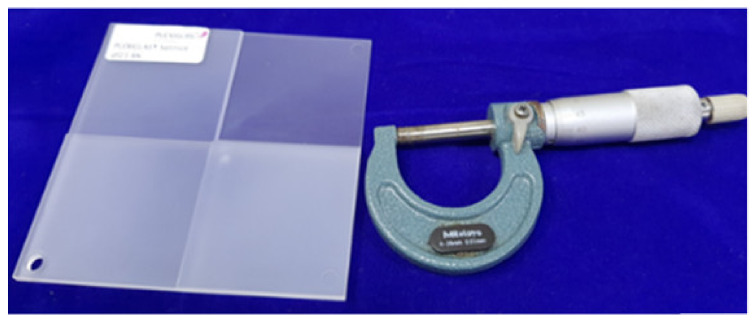
Sensitivity verification under different thicknesses (1 mm, 2 mm, 3 mm, and 4 mm) of IVI Bezel thickness Plaque.

**Figure 12:**
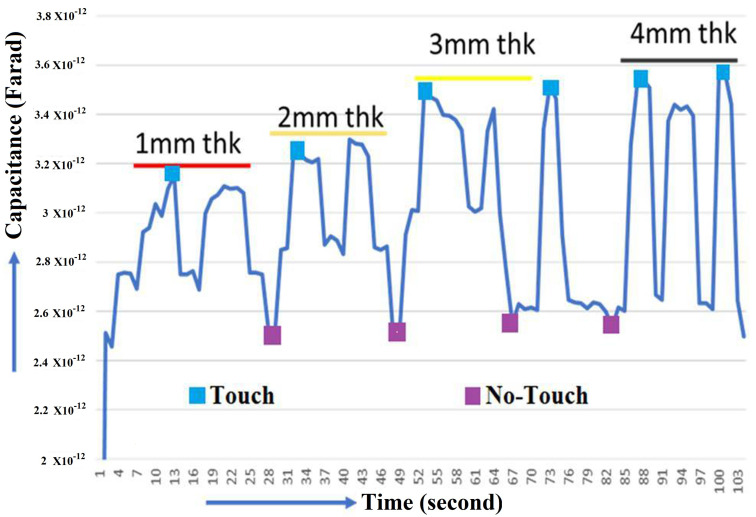
Change in capacitance with respect to thickness of IVI Bezel.

According to the findings of this study, the response parameters included the change in capacitance (C) during touch (pF) in order to measure the change in capacitance. The Keysight U1733C-20000 Counts Handheld LCR Meter with dual Display was used for the same. In the present study, the experimental trials with performance measures were designed in accordance with the design shown in Table 1. In order to improve measurement accuracy, all trials were carried out three times, with the average of the three values being used as the final value. The sensor is going to be mounted to a structural element (IVI Bezel) using an injection molding procedure. The sensor can be mounted at the top or bottom of the IVI bezel, depending on styling requirement. The direct (sensor on top) configuration may result in wear and tear. Hence the sensor on the bottom has been suggested. Its geometry and stylistic requirements determine the thickness of the IVI bezel, which can range from 1.5 mm to 3 mm in most cases. As a result, 1, 2, 3, and 4 mm as the IVI bezel thicknesses for touch sensitivity confirmation been selected as shown in the Figure 11. The Touch sensitivity was measured with Overlap 15 mm/0.5 mm sensor and noticed that the capacitance is increased with respect to thickness increase as shown in the Figure 12.

## 7. Conclusions

The purpose of this work was to develop an interdigitated pattern printed Ag electrode flexible sensor and a method for installation of sensor in the IVI Bezel for enhanced vehicle infotainment applications also performed the adhesive test as per automotive standard and finally the sensor is demonstrated the function of sensor with touch IC. The optimization technique was used to derive more advantageous design considerations and to examine the effect of sensor parameters on capacitance change during touch. On the basis of observation, the following findings were drawn.

➢ The interdigitated pattern with Ag nanoparticles conductive tracks and polycarbonate printed sensors provides significant sensing capability. The interdigitated design with overlap distance (15 mm) and electrode line width (0.5 mm) results in a greater change.
➢ The adhesion of the coating complies with the ISO and OEM requirement.
➢ The sensor can be used as a Class A or B surface
➢ The dashboard can be designed to be rugged, waterproof, and futuristic, such as a Pillar-to-Pillar cockpit, to meet the high-quality requirements of automotive applications.
➢ Due to optimal spatial interpolation, the overlap has a greater influence on the sensor’s performance.

However, additional study can be conducted using thermoforming process different 3D forms to meet the OEM surface requirements and also Electrode Line width of Tx and Electrode Line width of Rx are constant and Gap of both electrode with different gaps.

## Figures and Tables

**Table 1:** Computation of Sensor performance measures.

Overlap Distance	Electrode Line Width	Change in Capacitance ΔC
5	0.5	1.47
5	0.8	0.48
5	1.2	0.99
10	0.5	2.66
10	0.8	1.75
10	1.2	0.9
15	0.5	3.23
15	0.8	2.49
15	1.2	1.68
